# Clinical Interest of LMO2 Testing for the Diagnosis of Aggressive Large B-Cell Lymphomas

**DOI:** 10.3390/cancers12040884

**Published:** 2020-04-05

**Authors:** Ivonne Vazquez, Natalia Papaleo, Eugenia Garcia, Marta Salido, Antonio Salar, Silvia Hernandez, Xavier Calvo, Luis Colomo

**Affiliations:** 1 Department of Pathology, Hematopathology Section, Hospital del Mar, Institute of Investigacions Mediques-IMIM, Universitat Autonoma de Barcelona, 08003 Barcelona, Spain; ivazquez@psmar.cat (I.V.); npapaleo@psmar.cat (N.P.); msalido@psmar.cat (M.S.); xcalvo@psmar.cat (X.C.); 2 Department of Pathology-IdiPAZ, Hospital Universitario La Paz, 28046 Madrid, Spain; euge17@yahoo.com; 3 Department of Hematology, Hospital del Mar, Institute of Investigacions Mediques-IMIM, Universitat Autonoma de Barcelona, 08003 Barcelona, Spain; Asalar@psmar.cat; 4 Department of Health and Experimental Sciences, Universitat Pompeu Fabra, 08003 Barcelona, Spain; silvia.hernandez@upf.edu

**Keywords:** *MYC* rearrangement, LMO2, large B-cell lymphoma, immunohistochemistry

## Abstract

*MYC* rearrangements usually confer aggressive biological behavior to large B-cell lymphomas. In this study, we aimed to evaluate the relevance of LMO2 detection to the clinical approach to these tumors. First, the ability of LMO2 loss of expression to recognize the presence of *MYC* rearrangements was evaluated. A series of 365 samples obtained from 351 patients, including 28 Burkitt lymphoma, 230 diffuse large B-cell lymphoma, 30 high-grade B-cell lymphoma with *MYC* and *BCL2/BCL6* rearrangements, eight high-grade B-cell lymphoma-NOS, 43 transformed diffuse large B-cell lymphoma, and 26 high-grade follicular lymphomas was analyzed. Among the CD10-positive tumors prospectively analyzed in whole tissue sections, LMO2 negative expression obtained values of 88% sensitivity, 94% specificity, and 93% accuracy, proving the utility of LMO2 to screen *MYC* rearrangements. In addition, survival analyses were performed in a series of 155 patients. As per univariate analyses, the prognosis relevance of LMO2 was as useful as that of the diagnostic categories, *MYC* rearrangements, and MYC immunohistochemistry. Multivariate models revealed that both LMO2 (hazard ratio 0.51 *p* = 0.02) and IPI (hazard ratio 1.67 *p* < 0.005) were independent variables predicting overall survival. Finally, *MYC* and *LMO2* mRNA expression were analyzed in a small group of cases. Taken together, these findings show the interest of LMO2 testing in large B-cell lymphomas.

## 1. Introduction

MYC protein belongs to the family of MYC transcription factors, that in humans includes N-MYC and L-MYC [1,2]. They share similar structures and functions but have distinct targets and different expression patterns. MYC regulates multiple pathways orchestrating a broad spectrum of genes involved in the regulation of the cell cycle, DNA replication and cell division, cell growth, metabolism, protein biosynthesis, and differentiation. Additionally, MYC may cause genomic instability and is involved in the induction of apoptosis controlling BCL-2 protein family members and the p53 tumor suppressor pathway [2,3]. DNA damage caused by MYC overexpression has been associated with telomere aggregation and chromosome remodeling, resulting in loss of chromosomal integrity and therefore facilitating chromosome rearrangements. Moreover, the overexpression of MYC induces sustained DNA damage response and delays double-strand breaks DNA repair [4,5].

*MYC* is commonly dysregulated in aggressive B-cell lymphomas by gene rearrangements, amplifications or mutations. *MYC* rearrangements occur in 5–15% of diffuse large B-cell lymphomas (DLBCL), 20–35% of high-grade B-cell lymphoma, NOS (HGBL-NOS), and 90% of Burkitt lymphoma (BL). Moreover, the presence of such genetic alteration is a defining criterion of the HGBL category with rearrangements of *MYC* and *BCL2* and/or *BCL6* (HGBL-DH/TH) [6]. Thus, the identification of this specific gene alteration is of relevance for the diagnosis and prognosis of most of these entities. The best approach to identify *MYC* rearrangements is not determined yet. In clinical practice, fluorescence in situ hybridization (FISH) and classic G-banding cytogenetics are the most commonly used techniques to detect chromosomal alterations. Metaphase karyotyping requires fresh tissue and cell cultures with dividing cells, which are often neither feasible nor available in laboratories. In addition, this technique may miss some *MYC* cryptic rearrangements identified by FISH [7]. Interphase FISH using break-apart probes has become the most common approach used to identify *MYC* rearrangements. However, some studies have shown that this approach may not recognize 4–10% of cases with *MYC* rearrangements, and those cases could just be detected using dual fusion probes [8,9]. Cryptic rearrangements of *MYC* have been recently identified in cases of DLBCL with unfavorable prognosis. Those cases shared similar gene expression profile like HGBL-DH/TH and were not recognized by conventional FISH methods [10]. Thus, the global incidence of *MYC* rearrangements in large B-cell lymphomas (LBCL) being low, it is necessary to clarify whether FISH or other methods have to be applied to all LBCL or just in selected cases. Furthermore, provided such approaches cannot be afforded by all laboratories, the research of useful surrogate markers is required to screen *MYC* rearrangements.

The *LMO2* (hematopoietic transcription factor LIM domain only 2) gene was initially described as a recurrent chromosomal translocation partner of the TCR gene in a subset of patients with T-cell acute lymphoblastic leukemia. With the development of gene expression profile technology, *LMO2* arose as an important gene defining the germinal center B-cell (GCB) molecular subgroup of DLBCL, as well as a relevant prognostic marker in DLBCL [11,12,13]. Recent studies have shown that LMO2 protein expression in DLBCL induces genomic instability [14,15]. We previously observed that *LMO2* gene expression was frequently downregulated in cases with *MYC* rearrangements and identified that LMO2 loss of protein expression captured better than MYC expression most of those cases [16]. In this study we corroborate the clinical utility of our previous observations adding new data recording the relationship between LMO2 and MYC.

## 2. Results

### 2.1. MYC Protein Expression and Gene Rearrangements in Aggressive B-Cell Lymphomas

We analyzed a series of 365 samples from 351 patients with LBCL including 28 cases diagnosed of BL, 230 DLBCL, 30 HGBL-DH/TH, eight HGBL-NOS, 43 transformed low-grade lymphomas into DLBCL (tDLBCL; 39 transformed follicular lymphomas, three transformed marginal zone lymphomas, and one transformed lymphoplasmacytic lymphoma) and 26 grade 3A follicular lymphomas (FL), diagnosed according to WHO criteria [6].

Table 1 shows clinical features of the patients, and immunohistochemical and genetic alterations of cases with available data. The incidence of the most common markers such as CD10, BCL6, and MUM1, detected by immunohistochemistry (IHC), was similar to a previous published series [17,18]. Among 221 DLBCL with available information, 94 (42%) cases had a GCB-like IHC profile, and 127 were non-GCB following the Hans algorithm [19]. Twenty-seven out of 30 (90%) HGBL-DH/TH, and 7/8 (88%) HGBL-NOS could be classified as GCB-like following the same approach.

MYC protein expression over 40% of neoplastic cells was present in 27/28 (96%) HGBL-DH/TH and 15/20 (75%) BL, all of them carrying *MYC* rearrangements, and 7/8 (87%) HGBL-NOS. In the latter group, 6/7 *MYC* rearranged cases expressed MYC as well as one case lacking *MYC* rearrangement. Interestingly, one patient that presented with pelvic mass and peripheral blood involvement at diagnosis, classified in the group of HGBL-NOS carrying *MYC* rearrangement, acquired an additional t(18;22) at relapse, being therefore classifiable as HGBL-DH/TH. 

The group of DLBCL included 69/220 (31%) MYC positive cases detected by IHC. Among the 69 MYC positive cases, 12 (17%) carried *MYC* rearrangements, whereas only two MYC negative cases out of 151 (1%) were *MYC* rearranged (*p* < 0.001). In tDLBCL, MYC was expressed in 13/40 (32%) cases. Among them, 10/13 (77%) were *MYC* rearranged cases, and only 1/27 (4%) MYC negative cases was *MYC* rearranged (*p* < 0.001). The high incidence of *MYC* rearrangements in this group can be attributed to the main definition of the category HGBL-DH/TH included the WHO classification, that excludes cases of proven follicular lymphoma. Among tDLBCL with FL and *MYC* and *BCL2/BCL6* rearrangements, five patients had previous history of FL, and four had simultaneous DLBCL and FL in the same biopsy. In three of four cases included in the latter group, MYC was expressed in both components, although *MYC* rearrangement was identified only in the DLBCL component. Of note, in none of grade 3A FL cases with available information MYC was expressed or rearranged.

In 45 cases we could determine the partner of MYC rearrangements. Table 1 shows 36 cases *MYC/IG*-rearranged (34 *IGH*, one HGBL-DH/TH, and 1BL were *IG-lambda*, the latter cases identified by conventional karyotyping) and nine cases *MYC/non-IG*. Five out of eight (62%) tDLBCL and 3/15 (20%) HGBL-DH/TH were *MYC/non-IG* (*p* = 0.013). 

### 2.2. LMO2 Is Downregulated in MYC-Rearranged Aggressive B-Cell Lymphomas

LMO2 protein was negative in 21 BL studied, had low incidence of expression in the categories associated with *MYC* rearrangements (HGBL-DH/TH and HGBL-NOS, 27% and 25%, respectively), and was frequently expressed in FL, tDLBCL, and DLBCL (100%, 70%, and 63%, respectively). The statistical analyses showed significant statistical association between the loss of expression of LMO2 and the presence of *MYC* rearrangements in the whole series of cases (*p* < 0.005). To avoid the potential bias caused by an overrepresentation of CD10-positive cases, BL and FL grade 3A were excluded of the analyses and significant results were obtained in 272 analyzed cases (*p* < 0.005).

To assess whether LMO2 could be a useful marker to screen *MYC* rearrangements, prospective and retrospective cases were studied. For this purpose, in prospective cases all markers were evaluated in whole tissue sections (WTS), as during the work up of cases. To analyze retrospective cases, WTS and or tissue microarrays (TMAs) were used. The statistical analyses were carried out according to these two groups. The statistical analysis showed significant correlations between LMO2 expression and *MYC* rearrangements in both prospective and retrospective groups (*p* < 0.005 for both groups). Table 2 shows the statistic measures of the performance of LMO2 and MYC compared with the presence of *MYC* gene rearrangements as gold standard.

The results are very similar to those obtained in our previous study, that included a different series of patients [16]. Thus, LMO2 protein is a marker with similar sensitivity, specificity, accuracy, positive predictive value (PPV), negative predictive value (NPV), and likelihood ratios (LR) as MYC protein, particularly in those studied prospectively. Moreover, in CD10-positive cases the loss of LMO2 expression captures better the presence of *MYC* rearrangements than MYC protein expression, since they occur more frequently in GCB-derived aggressive large B-cell lymphomas (Table 2 and Table S1). When we used the Hans algorithm to classify the cell of origin instead of CD10 expression, we observed lower specificity (77% vs. 94%), PPV (56% vs. 81%), accuracy (80% vs. 93%), and positive LR (3.88 vs. 14.96) in GCB-like cases, compared with CD10-positive cases. The sensitivity was 88% for both approaches, and the NPV and negative LR were 95% vs. 96%, and 0.16 vs 0.13, respectively.

### 2.3. LMO2 Captures the Impact of Known Prognostic Factors in Aggressive LBCLs

We performed survival analyses assessing the clinical impact of LMO2 in a series of 155 LBCL patients with available clinical information including: seven FL g3A, 22 tDLBCL, 105 DLBCL, 20 HGBL-DH/TH, and four HGBL-NOS.

**Figure 1:**
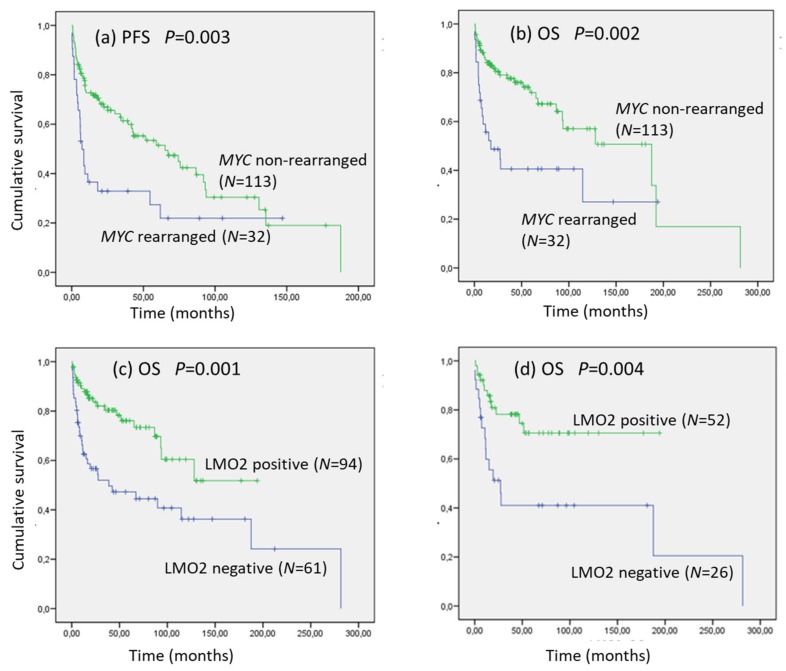
Progression-free survival (PFS) (**a**) and overall survival (OS) (**b**) of MYC rearrangements in 155 LBCL. OS of LMO2 expression in 155 LBCL (**c**) and 78 CD10-positive LBCL (**d**).

Survival features are detailed in Table 3 and plotted in Figure 1. According the diagnostic categories, the 5-year progression-free survival (PFS) was 25% for tDLBCL and HGBL-NOS, (median PFS 25.05 and 11.19 months, respectively), 55% for DLBCL, and 20% for HGBL-DH/TH (*p* = 0.009). In addition, the 5-year PFS was significantly lower for the presence of *MYC* rearrangement (23% vs. 51%, *p* = 0.001), MYC IHC expression (32% vs. 51%, *p* = 0.001), and LMO2 loss of expression (33% vs. 53%, *p* = 0.05).

The 5-year overall survival (OS) according the diagnostic categories was 76% for tDLBCL, 68% for DLBCL, 50% for HGBL-NOS, and 25% for HGBL-DH/TH (*p* = 0.003). Moreover, the 5-year OS was significantly shorter for the presence of *MYC* rearrangement (38% vs. 72%, *p* = 0.002), MYC protein expression (47% vs. 74%, *p* = 0.001), and LMO2 loss of expression (46% vs. 76%, *p* = 0.001). All these significant results were obtained independently of the administered treatment and were also significant in 112 patients treated with curative intention (Table S2). As LMO2 captures better the presence of *MYC* rearrangements in CD10-positive LBCLs, we performed the survival analyses in this group, obtaining similar results as in the whole series of cases (Table 3).

Finally, in a Cox regression survival analysis including the International Prognostic Index (IPI) and LMO2 for 140 cases, IPI (HR: 1.67 *p* < 0.005) and LMO2 (HR: 0.51 *p* = 0.02) were the most important variables to predict OS. Moreover, the models including MYC IHC, *MYC* rearrangements, and diagnostic category did not add predictive accuracy to IPI score (HR: 1.45 *p* = 0.32; HR: 0.95 *p* = 0.89; HR: 1.28 *p* = 0.27, respectively). These results were similar considering CD10-postive cases in 45 patients treated with curative intention (IPI HR: 2.46 *p* = 0.01).

### 2.4. Interest of MYC/LMO2 mRNA Expression and MYC/LMO2 Dissociated Cases

**Figure 2:**
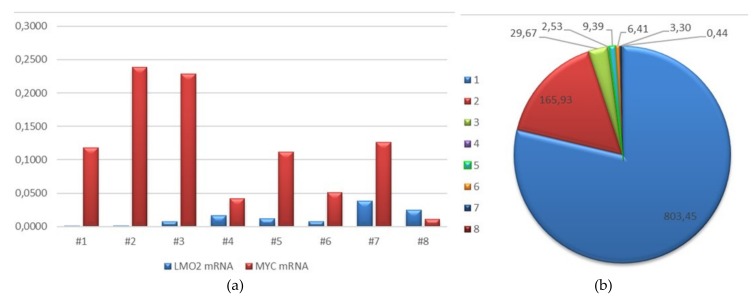
*MYC* and *LMO2* mRNA expression. (**a**) *LMO2* and *MYC* mRNA by quantitative real-time PCR (qPCR) in HGBL-DH/TH (cases #1 to #4), tDLBCL with *MYC* rearranged, (#5, #6), tDLBCL with *MYC* amplified (#7), and DLBCL with *MYC* gained (#8); (**b**) *MYC* mRNA:*LMO2* mRNA in same cases.

The mRNA expression of *MYC* and *LMO2* was studied by quantitative real-time PCR (qPCR) in eight cases. The results are shown in Figure 2 and Table S3. Cases #1 to #4 were HGBL-DH/TH, #5 and #6 tDLBCL with *MYC* rearrangements (both FL), #7 a tDLBCL with *MYC* amplification, and #8 DLBCL with *MYC* gains and *BCL2* rearrangement. To evaluate the relationship between the expression of both genes a ratio *MYC:LMO2* was obtained for each case. Cases #1 to #3 had the highest ratios (range 29.7–803.4) and patients had an OS between 1 and 12 months. Conversely, patients with low ratios *MYC:LMO2* (range 2.5–9.3) were long survivors and all are still alive (range 87–194 months) even carrying double or triple rearrangements of *MYC* and *BCL2/BCL6*. These preliminary results raise the interest of the detection of *MYC* and *LMO2* mRNA expression and warrant additional studies.

**Figure 3:**
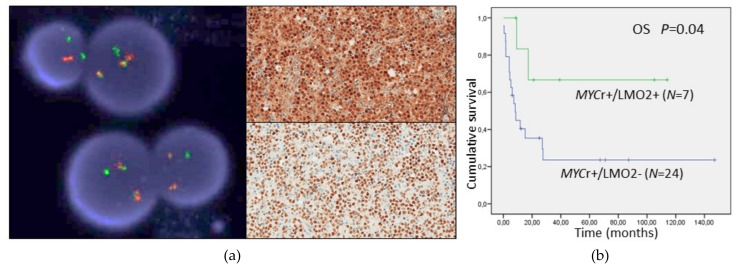
(**a**) An example of dissociated case, with *MYC* rearranged and high expression of LMO2 protein (up) and MYC protein (down) (case #5 from Figure 2); (**b**) OS comparing *MYCr*+/LMO2+ and *MYCr*+/LMO2− cases.

CD10 identifies GCB-derived cases and LMO2 is frequently downregulated in this group when *MYC* is rearranged. However, in our previous study we found CD10-positive LBCL cases with *MYC* rearrangement and LMO2 expression (*MYCr*+/LMO2+), that we defined as “dissociated” cases (DC). Such group did not present relevant clinicopathological differences compared with non-DC cases with *MYCr*+/LMO2- profile [16]. In the present study we wanted to know the clinical behavior of DC. Eight samples from eight patients met this criterion: three tDLBCL (all transformed FL) and five HGBL-DH/TH. Clinical information was available in eight DC and 26 non-DC. Among 31 patients receiving treatment, the 5-year OS was 67% for DC cases and 21% for non-DC (*p* = 0.04) (Figure 3). Among 23 patients receiving treatment with curative intention, 5/6 (83%) *MYCr*+/LMO2+ achieved complete remission (CR) compared with 6/17 (35%) *MYCr*+/LMO2− patients (*p* = 0.04). The 5-year OS in this group was 60% for DC cases and 35% for non-DC (*p* = 0.2).

## 3. Discussion

In the present study we aimed to evaluate the clinical utility of LMO2 protein expression for the diagnostic approach of LBCL. First, we assessed the ability of LMO2 loss of expression to capture the presence of *MYC* rearrangements. Thus, we used a similar approach to evaluate the reproducibility of the results obtained in our previous study, based on 330 patients. In the previous survey, we observed a value of 87% for the sensitivity, specificity, and accuracy, respectively, for the detection of *MYC* rearrangements when LMO2 was downregulated in CD10-positive tumors [16]. Now, we analyzed a series of 365 cases, obtaining values of 88%, 94%, and 93% for the same measures, respectively, reinforcing our previous observations. In the present study, we also analyzed our results in prospective and retrospective cases. Therefore, we compared the immunohistochemical results in WTS in the former group, with those obtained in WTS and TMAs in the latter. Better results were obtained in the prospective group, indicating the benefit of including LMO2 as diagnostic marker in the workflow of LBCL. Thus, these intriguing results indicate that LMO2 may be a useful marker to screen *MYC* rearrangements in aggressive LBCL, particularly in CD10-positive cases. Further studies should evaluate whether this approach may be useful to screen cases carrying FISH-cryptic rearrangements of *MYC*.

Another objective was to evaluate whether LMO2 behaves as several proven prognostic factors in LBCL, such as the classification based on clinicopathological diagnostic entities and categories, the clinical IPI, the presence of *MYC* rearrangements, and MYC protein expression detected by immunohistochemistry. The survival analyses performed for all these variables offered significant results and indicates that LMO2 captures the significant prognostic impact of these proven variables as well. Several published studies have shown the prognostic impact of LMO2 at different levels. Gene expression profiling (GEP) studies identified *LMO2* as one of the relevant genes defining the GCB-like signature in DLBCL. As known, this group associates with a favorable outcome both in both pre- and Rituximab era [11,20,21]. To ease the translation to clinics of GEP analyses, selected panels including reduced number of markers were defined, and some studies demonstrated the prognostic impact of *LMO2* gene expression in this setting [12,22]. At the protein level, LMO2 was included in the Tally algorithm as one of the proteins defining the GCB-like immunohistochemical profile [23]. As a single prognostic marker, LMO2 protein expression had also shown prognostic impact on DLBCL [13]. In our study, we obtained an independent prognostic impact for LMO2 in the multivariate analyses, as observed in the study of Natkunam et al., enhancing the interest in studying LMO2 protein by immunohistochemistry in LBCL [13].

In addition, some suggestive ideas arise from the present study, such as the interest of testing MYC expression in FL, and the differences in the survival of cases carrying DH-TH. Thus, MYC expression was observed in three of four cases of composite FL and DLBCL carrying *MYC* rearrangements, whereas MYC protein expression was negative in all grade 3A FL studied. As *MYC* genetic alterations only appeared in composite FL and DLBCL, it seems to make sense to explore MYC protein expression in this clinical situation to screen *MYC* rearrangements. On the other hand, we observed remarkable differences in survival of patients with LBCL with low *MYC:LMO2* ratios, compared with cases with higher ratio. Of note, two of three long survivors corresponded to tDLBCL from FL. Miyaoka et al. compared the clinicopathological features and genomic complexity of FL carrying *MYC* rearrangements (DH-FL) with HGBL-DH/TH [24]. The biological behavior was more favorable for patients with DH-FL, and the genomic complexity was lower in such cases. Favorable outcomes of DH-FL were also observed in other studies in patients treated with conventional or intensive schemes [25,26,27]. Recent studies have defined the molecular high-grade B-cell lymphoma profile (MGH) by gene expression and mutational analysis [28,29]. These studies identified cases that did not carry *MYC* rearrangements and behaved aggressively. The authors also described the contrary situation, identifying HGBL-DH/TH with similar behavior as GCB-DBCL. *MYC* mRNA levels play a remarkable role in MGH profile, suggesting the validity of our preliminary results assessing *MYC:LMO2* ratios.

LMO2 appears as a helpful marker to identify BL, and it is usually negative in this entity, as observed in the present and our previous study [16]. Therefore, these results improve MYC IHC for the characterization of BL, which sometimes may lack MYC protein expression even carrying *MYC* translocations. In our series, 3/30 [16] and 5/20 BL cases lacked MYC expression. Some studies showed that *MYC* mutations are common in BL and mainly locate in the first 100 aa of the MYC protein. This is the region recognized by MYC clone Y69, and this may explain the absence of MYC protein expression in such cases [30]. Moreover, recent studies suggest that LMO2 also appears to be useful for the differential diagnosis of BL and the provisional category of Burkitt-like lymphoma with 11q aberration. Two independent studies characterizing Burkitt-like lymphoma with 11q aberration, noted LMO2 protein expression in 7/10 (70%) and 5/11 (46%) cases [31,32]. An additional study including 75 BL observed absent expression of LMO2 in 74 (99%) cases, whereas three out of three Burkitt-like lymphomas with 11q aberration were positive [33]. Taken together, these results support our observations regarding the low levels of protein expression in BL and its correlations with *MYC* rearrangements.

Finally, recent studies associate LMO2 protein expression with genomic instability in DLBCL. Cubedo et al. showed a link between *LMO2* gene overexpression and the expression of genes related to chromosomal assembly and segregation in mitosis, to DNA damage, and response to cellular stress, resulting in genomic instability [14]. Recently, Parvin et al. demonstrated that DLBCL expressing LMO2 protein are functionally deficient in homologous recombination (HR)-mediated double strand breaks (DSB) DNA repair [15]. In this study, the authors identified the impairment of the HR pathway in LMO2 positive tumors and how this alteration sensitized such DLBCL to inhibitors of the preserved pathways. MYC also impairs the mechanisms of DSB repair. As LMO2 expression determines diverse biological behavior in LBCLs, a hypothesis to be assessed might be whether *MYC* rearranged LBCLs downregulate LMO2 to gain survival advantage.

## 4. Materials and Methods

### 4.1. Case Selection

We studied two series of patients diagnosed of LBCL, including BL, grade 3A FL, DLBCL, HGBL-DH/TH, HGBL-NOS, and tDLBCL, diagnosed according to WHO criteria [6]. Primary mediastinal large cell lymphoma, T-cell rich B-cell lymphoma, HHV8-associated lymphoma, and plasmablastic lymphoma or transformed myeloma were not included in the study. The first series included 365 samples from 351 patients and evaluated the relationship between LMO2 expression and *MYC* rearrangements. Among patients with more than one sample, four patients experienced transformation from FL to DLBCL, and the other patients did not change the first diagnosis during the course of the disease. This series included consultation cases and cases received at our institution for cytogenetic studies. All cases were diagnosed between 2000 and 2019, with the criterion of the availability of adequate histological material. The series used for survival analysis included 155 patients diagnosed and treated at Hospital del Mar. The study was approved by the Ethics Committee of the Hospital del Mar of Barcelona (2017/7481/I). Informed consent to use both clinical data and histological material was obtained in accordance with the Declaration of Helsinki.

### 4.2. Immunohistochemistry

Cases were classified as prospective and retrospective if diagnosed after or before September 2014. The prospective cohort included 215 cases studied in whole tissue sections (WTS). The retrospective cohort included 150 cases, 34 of them studied using WTS and 116 in TMAs, which included two 1-mm representative cores of each case and were constructed using a tissue arrayer (MTA I; Beecher Instruments Inc, Sun Prairie, WI. USA). Immunohistochemical studies were performed using a panel of monoclonal antibodies reactive in paraffin-embedded tissue sections using a peroxidase-labeled detection system, standard antigen retrieval protocols, and automated immunostainer (Benchmark XT, Ventana, Roche, Tucson, AZ, USA). Standard methods for tissue fixation (10% buffered formalin) and processing were used. The panel of antibodies included common B- and T-cell markers, BCL2 (clone 124), CD10 (clone SP67), BCL6 (clone GI191E-A8), MUM1/IRF4 (clone MRQ-43), Ki-67 (clone MIB-1), and MYC (clone Y69). The conditions and the evaluation for all these antibodies were the same as previously described and were assessed as previously described, using appropriate internal and external controls [16]. The immunohistochemical study included the identification of the cell of origin (COO) for DLBCL cases by the Hans algorithm [19]. The cutoff for MYC immunostaining was 40%, as reported [16,17]. LMO2 (clone 1A9-1, Ventana-Roche, USA) was incubated for 16 min, after the antigen retrieval with CC1 solution for 16 min and detected by OptiView Universal DAB Detection Kit in an automated immunostainer (Benchmark XT, Ventana, Roche, Tucson, AZ, USA). A cutoff of 30% was assigned for LMO2.

### 4.3. Fluorescence In Situ Hybridization (FISH)

FISH was performed and evaluated as previously described, following the criteria of Ventura [34]. Break-apart probes (BAP) specific for *MYC* (8q24) were applied in all cases included in the study. A dual fusion probe specific for *IGH/MYC/CEP8* was used in 45 cases with *MYC* rearrangement identified with BAP probes. For *BCL2* dual fusion probes *IGH/BCL2* were used, whereas BAP probes were performed for *BCL6*, as previously described, all from Vysis (Abbott Molecular, Des Plainescity, IL, USA) [16]. The cut-off values for the interphase FISH analyses were established following the criteria of Ventura, and the criteria for gains and amplifications were the same as reported [18].

### 4.4. LMO2 and MYC Quantitative mRNA Expression Analysis (qPCR)

Total RNA was extracted from eight frozen lymphoma samples with Ultraspec and the RNeasy Mini kit (Qiagen, Cathsworth, CA, USA) from 8–10 sections of 10 µm. RNA purity and quality were assessed with the Nanodrop^®^ ND-100 spectrophotometer (Nanodrop Technologies, Wilmington, DE, USA) and the Agilent 2100 Bioanalyzer (Agilent, Santa Clara, CA, USA). cDNA was synthesized using 1 µg of total RNA and Superscript IV Kit (Invitrogen, Thermo Fisher Scientific, Carlsbad, CA, USA).

*LMO2* and *MYC* mRNA expression were analyzed by quantitative Real-Time PCR (qPCR) in all samples, with the ABI PRISM 7500 Sequence Detection System, using the TaqMan^®^ Gene Expression Assay probe and primer mix (Applied Biosystems, Life Technologies Corporation, Foster City, CA, USA). The assay identification number for *LMO2* and *MYC* were Hs00153473_m1, and Hs00153408_m1, respectively. The *GAPDH* (4310884E) gene was used as internal control to normalize levels of mRNA expression (2^(−∆Ct)^). The samples were run in triplicate, the mean value was calculated, and a ratio between *MYC* and *LMO2* was obtained in each case.

### 4.5. Statistical Analysis

Data were compared using Chi-square test, unpaired *t*-tests, or nonparametric tests when necessary. *p* values < 0.05 were considered statistically significant for all tests. Accuracy, sensitivity, specificity, and positive and negative predictive values of LMO2 were calculated for 360 patients with LBCL. Likelihood positive and negative ratios were calculated to evaluate diagnostic accuracy. Standard definitions of complete response, progression-free survival, and overall survival were used, and survival analysis was carried out according to the method described by Kaplan and Meier and the curves compared by the log-rank test. The multivariate analyses for survival were performed using the stepwise proportional hazards model (Cox) as previously described [18].

## 5. Conclusions

In summary, in this study we show the interest of LMO2 testing in aggressive LBCL. We confirm the utility of LMO2 to define the profile of BL and the relevance of LMO2 as a surrogate marker for the detection on *MYC* rearrangements, particularly among CD10-positive tumors. In addition, we show that LMO2 captures the prognostic impact of other known markers and show the importance of this marker for the prognosis of these lymphomas. Otherwise, in the study we are recording the interest of *MYC* mRNA detection and the potential interest of *MYCr*/LMO2 profile to delineate the biological behavior of cases carrying *MYC* rearrangements. Finally, a potential relationship between the presence of *MYC* rearrangements, LMO2 expression, and genomic instability arises from this study.

## Figures and Tables

**Table 1:** Clinical and immunohistochemical features, and *MYC* gene alterations detected by fluorescence in situ hybridization (FISH) in 365 cases of large B-cell lymphomas (LBCL).

Clinical Features	BL	FL g3A	tDLBCL	DLBCL	HGBL-DH/TH	HGBL-NOS
Number of cases	28	26	43	230	30	8
Median age *	30 (2–56)	61 (36–89)	64 (40–82)	66 (21–97)	67 (39–94)	63 (42–89)
Gender (male/female) *	15/13	16/9	20/19	138/84	19/10	3/5
Primary extranodal	20/28 (71%)	5/26 (19%)	11/43 (26%)	135/230 (59%)	18/30 (60%)	4/8 (50%)
IHC						
CD10+	28/28 (96%)	23/26 (88%)	31/43 (72%)	83/227 (37%)	25/30 (83%)	7/8 (87%)
BCL6+	25/25 (100%)	25/25 (100%)	41/43 (95%)	198/222 (89%)	27/30 (97%)	8/8 (100%)
MUM1/IRF4+	7/15 (47%)	1/22 (4%)	19/40 (44%)	173/218 (79%)	8/29 (28%)	3/8 (37%)
BCL2+	2/28 (7%)	19/26 (73%)	38/43 (88%)	173/214 (81%)	27/29 (93%)	3/8 (37%)
MYC+	15/20 (75%)	0/21 (0%)	13/40 (32%)	69/220 (31%)	27/28 (96%)	7/8 (87%)
LMO2+	0/21 (0%)	24/24 (100%)	28/40 (70%)	123/196 (63%)	8/28 (27%)	2/8 (25%)
FISH						
*MYC*-N	0/28 (0%)	21/26 (81%)	21/43 (49%)	162/230 (70%)	0/30 (0%)	1/8 (25%)
*MYC*-R	28/28 (100%)	0/26 (0%)	11/43 (26%)	15/230 (6%)	30/30 (0%)	7/8 (87%)
*MYC+IG/MYC-*R	7/7 (100%)	0/0 (0%)	3/8 (37%)	8/9 (89%)	12/15 (80%)	6/6 (100%)
*MYC*-G	0/28 (0%)	5/26 (19%)	10/43 (23%)	49/230 (22%)	0/30 (0%)	0/8 (0%)
*MYC*-A	0/28 (0%)	0/26 (0%)	1/43 (2%)	4/230 (2%)	0/30 (0%)	0/8 (0%)
*BCL2*-R	0/9 (0%)	15/26 (58%)	25/41 (61%)	30/193 (15%)	25/29 (86%)	0/8 (0%)
*BCL6*-R	0/9 (0%)	3/23 (13%)	13/39 (33%)	49/173 (28%)	12/28 (43%)	0/8 (0%)

* Based on 351 patients; IHC, immunohistochemistry; *MYC*-N, *MYC* non-rearranged; *MYC*-R, *MYC* rearranged; *MYC+IG*, *MYC* rearranged with immunoglobulin heavy or light chains; *MYC*-G, *MYC* gained; *MYC*-A, *MYC* amplification; *BCL2*-R, *BCL2* rearranged; *BCL6*-R, *BCL6* rearranged.

**Table 2:** Statistic measures of the performance of LMO2 and MYC compared with the presence of *MYC* gene rearrangements as gold standard in LBCL.

Measure	All Series		Prospective Series		All Series CD10+		CD10+ Prospective Series	
	LMO2 (*n* = 317)	MYC (*n* = 337)	LMO2 (*n* = 210)	MYC (*n* = 209)	LMO2 (*n* = 175)	MYC (*n* = 177)	LMO2 (*n* = 110)	MYC (*n* = 107)
**Sensitivity (%)**	80/87		86/86		79/84		88/84	
**Specificity (%)**	71/76		75/76		89/78		94/80	
**PPV (%)**	48/53		41/42		81/69		81/57	
**NPV (%)**	91/95		96/96		88/91		96/94	
**Positive LR**	2.79/3.69		3.49/3.64		7.19/3.89		14.96/4.31	
**Negative LR**	0.28/0.16		0.18/0.18		0.24/0.18		0.13/0.2	
**Accuracy**	74/79		77/78		85/81		93/81	

PPV, positive predictive value; NPV, negative predictive value; LR, likelihood ratio.

**Table 3:** Clinical features and survival of patients with aggressive LBCL.

Clinical Features	All Cases (*n* = 155)	CD10+/LMO2- (*n* = 78)
Median age	67 (27–89)	67 (38–89)
Gender (male/female)	89/66	41/37
Diagnosis		
FL g3A	7/155 (4.5%)	3/78 (4%)
tDLBCL	22/155 (14%)	17/78 (22%)
DLBCL	105/155 (68%)	42/78 (54%)
HGBL, NOS	4/155 (2.5%)	4/78 (5%)
HGBL-DH/TH	17/155 (11%)	12/78 (15%)
Stage III/IV	85/151 (88%)	43/78 (56%)
IPI high (3–4)	69/144 (88%)	32/70 (41%)
Complete response	102/155 (66%)	49/78 (63%)
5y PFS/OS		
FL g3A	NR/NR	NR/NR
tDLBCL	25/76	25/76
DLBCL	55/68	48/70
HGBL-NOS	25/50	25/50
HGBL-DH/TH	20/25	25/22
*MYC*-rearranged	23/38	23/39
MYC+ IHC	32/47	25 */48
LMO2− IHC	33/46	27 **/40

*p* < 0.05 for all survival results except * *p* = 0.067 and ** *p* = 0.251.

## Supplementary Materials

The following are available online at https://www.mdpi.com/2072-6694/12/4/884/s1, Table S1: CD10 versus *MYC* gene status according diagnostic categories in 362 cases of LBCL with available results, Table S2: Five year PFS and OS in 112 patients with aggressive LBCL treated with curative intention; Table S3: Clinicopathological features of patients with LMO2 and MYC mRNA results.
