# Supplementary material for: Clinical Interest of LMO2 Testing for the Diagnosis of Aggressive Large B-Cell Lymphomas
Source: Cancers (Basel). 2020 Apr 5;12(4):884. doi: 10.3390/cancers12040884 (PMC7226002; doi:10.3390/cancers12040884)
Supplement: Supplementary file 1 [file cancers-12-00884-s001.pdf]

cancers

MDPI

Article

Clinical Interest of LMO2 Testing for the Diagnosis of Aggressive Large B-Cell Lymphomas

Ivonne Vazquez, Natalia Papaleo, Eugenia Garcia, Marta Salido, Antonio Salar, Silvia Hernandez, Xavier Calvo and Luis Colomo

Table S1. CD10 versus *MYC* gene status according diagnostic categories in 362 cases of LBCL with available results.

	BL	FL g3A	tDLBCL	DLBCL	HGBL-DH/TH	HGBL, NOS
**CD10-positive cases (*N*) ***	28	23	31	83	25	7
***MYC*-R**	28/28 (100%)	0/23 (0%)	7/31 (23%)	10/83 (12%)	25/25 (100%)	6/7 (86%)
***MYC* non-R**	0/28 (0%)	23/23 (100%)	24/31 (77%)	73/83 (88%)	0/25 (0%)	1/7 (14%)
**CD10-negative cases (*N*) ***	0	3	12	144	5	1
***MYC*-R**		0/3 (0%)	4/12 (33%)	5/144 (3.5%)	5/5 (100%)	1/1 (100%)
***MYC* non-R**		3/3 (100%)	8/12 (67%)	139/144 (96.5%)	0/5 (0%)	0/1 (0%)

* ($p < 0.005$); *MYC*-R, *MYC* rearranged; *MYC* non-R, *MYC* non-rearranged.

Table S2. Five year PFS and OS in 112 patients with aggressive LBCL treated with curative intention.

	5 year PFS		5 year OS		5 year OS at transformation	
Diagnosis						
FL g3A	NR		NR		NR	
tDLBCL	19		73		71	
DLBCL	65		70		77	
HGBL, NOS	50		50		50	
HGBL-DH/TH	21	*p = 0.007*	28	*p = 0.015*	29	*p = 0.013*
MYC-rearranged	25		40		39	
MYC non-rearranged	57	*p = 0.007*	77	*p = 0.008*	79	*p = 0.006*
MYC +	36		52		45	
MYC -	60	*p = 0.001*	80	*p = 0.001*	80	*p = 0.001*
LMO2 -	37		49		44	
LMO2 +	59	*p = 0.047*	83	*p = 0.002*	81	*p = 0.003*

Table S3. Clinicopathological features of patients with *LMO2* and *MYC* mRNA results.

Case # Gender, age	Diagnosis	*LMO2* mRNA	*MYC* mRNA	*MYC*:LMO2	*MYC* status	Follow-up, months
#1 F 94	HGBL-DH/TH	0,0001	0,1180	803,45	R	DwD, 12
#2 M 54	HGBL-DH/TH	0,0014	0,2385	165,93	R	DwD, 1
#3 M82	HGBL-DH/TH	0,0077	0,2284	29,67	R	DwD, 8
#4 M 61	HGBL-DH/TH	0,0167	0,0423	2,53	R	AneD, 105
#5 M 68	tDLBCL	0,0119	0,1119	9,39	R	AneD, 194
#6 F 78	tDLBCL	0,0079	0,0508	6,41	R	AneD, 87
#7 M 48	tDLBCL	0,0383	0,1265	3,30	A	DwD, 12
#8 M 77	DLBCL	0,0246	0,0107	0,44	G	Dned *, 49

R, rearranged; A, amplified; G, gained; DwD, dead with disease; AneD, alive with no evidence of disease; * DneD, dead with no evidence of disease (dead of liver disease).

© 2020 by the authors. Licensee MDPI, Basel, Switzerland. This article is an open access article distributed under the terms and conditions of the Creative Commons Attribution (CC BY) license (http://creativecommons.org/licenses/by/4.0/).
